# Single-vesicle imaging quantifies calcium’s regulation of nanoscale vesicle clustering mediated by α-synuclein

**DOI:** 10.1038/s41378-020-0147-1

**Published:** 2020-06-29

**Authors:** Bin Cai, Jie Liu, Yunfei Zhao, Xiangyu Xu, Bing Bu, Dechang Li, Lei Zhang, Wei Dong, Baohua Ji, Jiajie Diao

**Affiliations:** 1 Department of Cancer Biology, University of Cincinnati College of Medicine, Cincinnati, OH 45267 USA; 2 Biomechanics and Biomaterials Laboratory, Department of Applied Mechanics, Beijing Institute of Technology, Beijing, 100081 China; 3 Key Laboratory of Medical Electrophysiology of Ministry of Education and Medical Electrophysiological Key Laboratory of Sichuan Province, Institute of Cardiovascular Research, Southwest Medical University, Luzhou, Sichuan 646000 China; 4 Institute of Biomedical Engineering and Health Sciences, Changzhou University, Changzhou, Jiangsu 213164 China; 5 Institute of Applied Mechanics, Department of Engineering Mechanics, Zhejiang University, Hangzhou, 310027 China; 6 Key Laboratory for Nonequilibrium Synthesis and Modulation of Condensed Matter (Ministry of Education), School of Science, Xi’an Jiaotong University, Xi’an, 710049 China; 7 Beijing Advanced Innovation Center for Biomedical Engineering, Beijing, 100191 China

**Keywords:** Biosensors, Optical sensors

## Abstract

Although numerous studies have shown that the protein α-synuclein (α-Syn) plays a central role in Parkinson’s disease, dementia with Lewy bodies, and other neurodegenerative diseases, the protein’s physiological function remains poorly understood. Furthermore, despite recent reports suggesting that, under the influence of Ca^2+^, α-Syn can interact with synaptic vesicles, the mechanisms underlying that interaction are far from clear. Thus, we used single-vesicle imaging to quantify the extent to which Ca^2+^ regulates nanoscale vesicle clustering mediated by α-Syn. Our results revealed not only that vesicle clustering required α-Syn to bind to anionic lipid vesicles, but also that different concentrations of Ca^2+^ exerted different effects on how α-Syn induced vesicle clustering. In particular, low concentrations of Ca^2+^ inhibited vesicle clustering by blocking the electrostatic interaction between the lipid membrane and the N terminus of α-Syn, whereas high concentrations promoted vesicle clustering, possibly due to the electrostatic interaction between Ca^2+^ and the negatively charged lipids that is independent of α-Syn. Taken together, our results provide critical insights into α-Syn’s physiological function, and how Ca^2+^ regulates vesicle clustering mediated by α-Syn.

## Electrophysiology: Calcium regulation of vesicle clustering mediated by alpha-synuclein

Calcium plays a critical role in the clustering of synaptic vesicles (which in a neuron store various neurotransmitters released at the synapse) induced by the protein alpha-synuclein, and different calcium concentrations exert different effects on such clustering. Alpha-synuclein is a key element in the development of Parkinson’s disease and other neurodegenerative conditions: it has been shown to interact with synaptic vesicles under the influence of calcium, but its physiological functions are poorly understood. Jiajie Diao of the University of Cincinnati College of Medicine, USA and colleagues used single-vesicle imaging to examine how calcium regulates vesicle clustering mediated by alpha-synuclein. They found that high calcium concentrations promoted vesicle clustering independent of alpha-synuclein, but low calcium concentrations inhibited vesicle clustering in an alpha-synuclein–dependent manner. The authors believe their findings provide insights into alpha-synuclein’s physiological function.

## Introduction

α-Synuclein (α-Syn), a presynaptic protein abundantly expressed throughout the central nervous system, is the hallmark of Parkinson’s disease, dementia with Lewy bodies, and other neurodegenerative diseases^1,2^. Found to aggregate in a nucleation-dependent manner, α-Syn forms cytotoxic amyloid oligomers and fibrils^3–8^. Recent studies have indicated that α-Syn not only binds to the highly curved membranes of synaptic vesicles, but also senses and regulates the curvature of those membranes, which immediately suggests that α-Syn is involved in synaptic vesicle trafficking and its exo- and endocytosis^9–11^. However, the physiological function of monomeric α-Syn remains unclear^9,12^.

In aqueous solution, α-Syn is an intrinsically disordered protein that binds to small synaptic vesicles via a conserved lipid-binding domain. When binding to a lipid membrane, the N terminus of α-Syn forms either an extended helix structure or two broken structures^13,14^. It has also been proposed that α-Syn acts as a bridge between membranes—for example, that two helices each bind to two different synaptic vesicles—the result of which is vesicle clustering^15^. At the same time, recent studies have suggested that calcium ions (Ca^2+^) can mediate the interaction between α-Syn and lipid membranes. For instance, Zhang et al. demonstrated that Ca^2+^ competitively binds to anionic lipids, which triggers the dissociation of α-Syn from membranes^16,17^. However, upon finding that Ca^2+^ increases α-Syn’s binding to anionic lipids via α-Syn’s C terminus, Lautenschläger et al^18^. proposed that the neutralization of negative charges on residues at the C terminus via the dynamic binding of positively charged Ca^2+^ facilitates α-Syn’s interaction with phospholipid membranes. They also showed that α-Syn and Ca^2+^ balance the electrostatic interaction of synaptic vesicles, which facilitates vesicle clustering^18^. Another recent study revealed that α-Syn amyloid oligomers, acting as multivalent nanoparticles, can cause hemifusion in negatively charged vesicles^19^. However, how Ca^2+^ regulates the interaction of α-Syn and lipids remains uncertain.

**Fig. 1:**
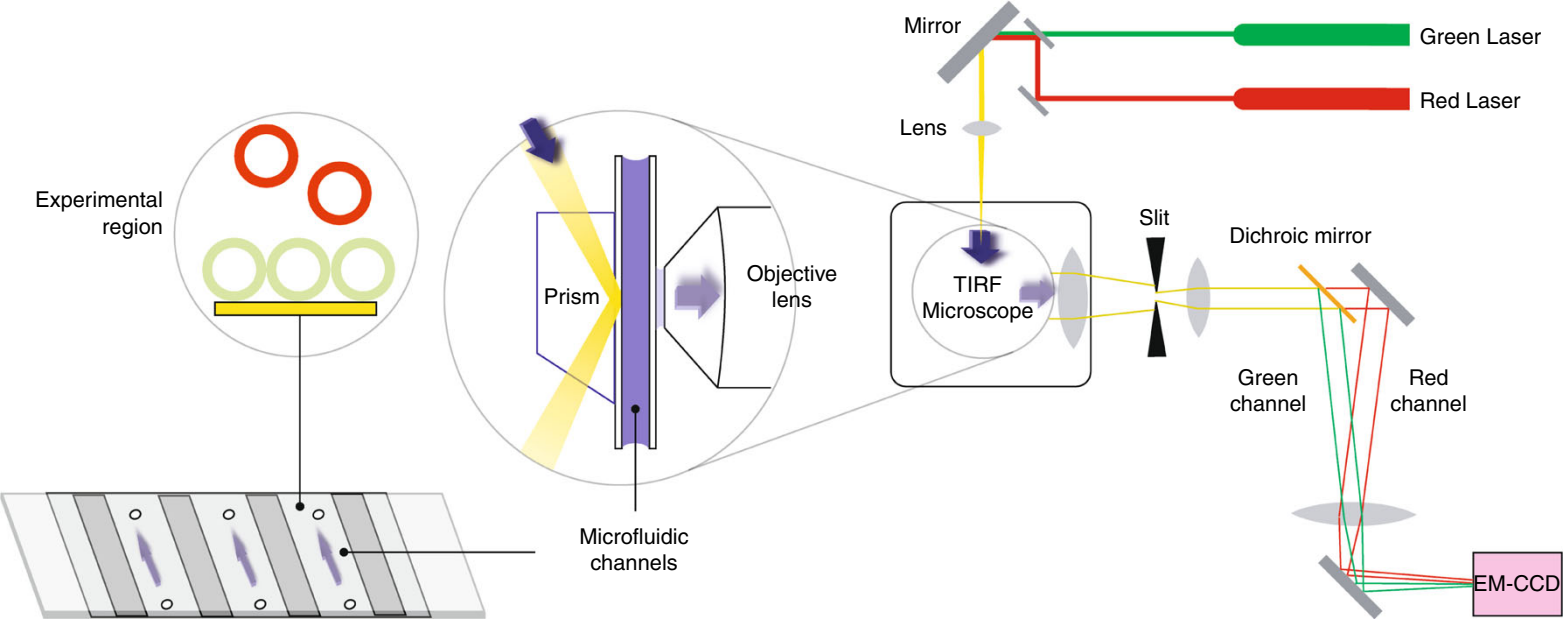
Illustration of the TIRFM system for studying the clustering of vesicles

Currently, researchers are in great need of a technique for studying the interactions between α-Syn and lipid membranes at the single-particle level^20^. As one option, total internal reflection fluorescence microscopy (TIRFM) is an optical technique for inducing an evanescent wave or field in a limited region of a specimen immediately adjacent to the interface between two media with different refractive indices^21,22^. A typical setup for TIRFM is shown in Fig. 1. Because the evanescent wave decays exponentially with distance from the interface’s surface, only fluorescent molecules within a few hundred nanometers of the interface are excited efficiently. In this way, TIRFM facilitates the excitation and observation of fluorophores within an extremely limited axial region^21,22^. Thus, TIRFM has benefited studies requiring images of individual particles in specimens with large numbers of fluorophores outside the optical plane of interest^21,22^, including during vesicle docking induced by proteins^23–26^.

In our study, we used single-vesicle imaging to quantify the extent to which Ca^2+^ regulates vesicle clustering mediated by α-Syn and to investigate the possible underlying mechanism. Among our results, α-Syn induced clustering of anionic lipid vesicles when no Ca^2+^ interaction occurred. Moreover, vesicle clustering induced by α-Syn was inhibited in the presence of low concentrations of Ca^2+^, where competitive binding of Ca^2+^ to lipids altered the electrostatic interaction and, in turn, caused the N terminus of α-Syn to dissociate from the membranes. By contrast, although Ca^2+^ inhibited the interaction of α-Syn and the lipid membranes, a high concentration of Ca^2+^ supplied sufficient multivalent ions to bridge vesicles into close contact and enhance vesicle clustering. Our results provide critical insights into α-Syn’s physiological function and the mechanism by which calcium ions regulate vesicle clustering mediated by α-Syn.

## Results

### Ca^2+^ regulates vesicle clustering mediated by α-Syn depending on Ca^2+^ concentration

**Fig. 2:**
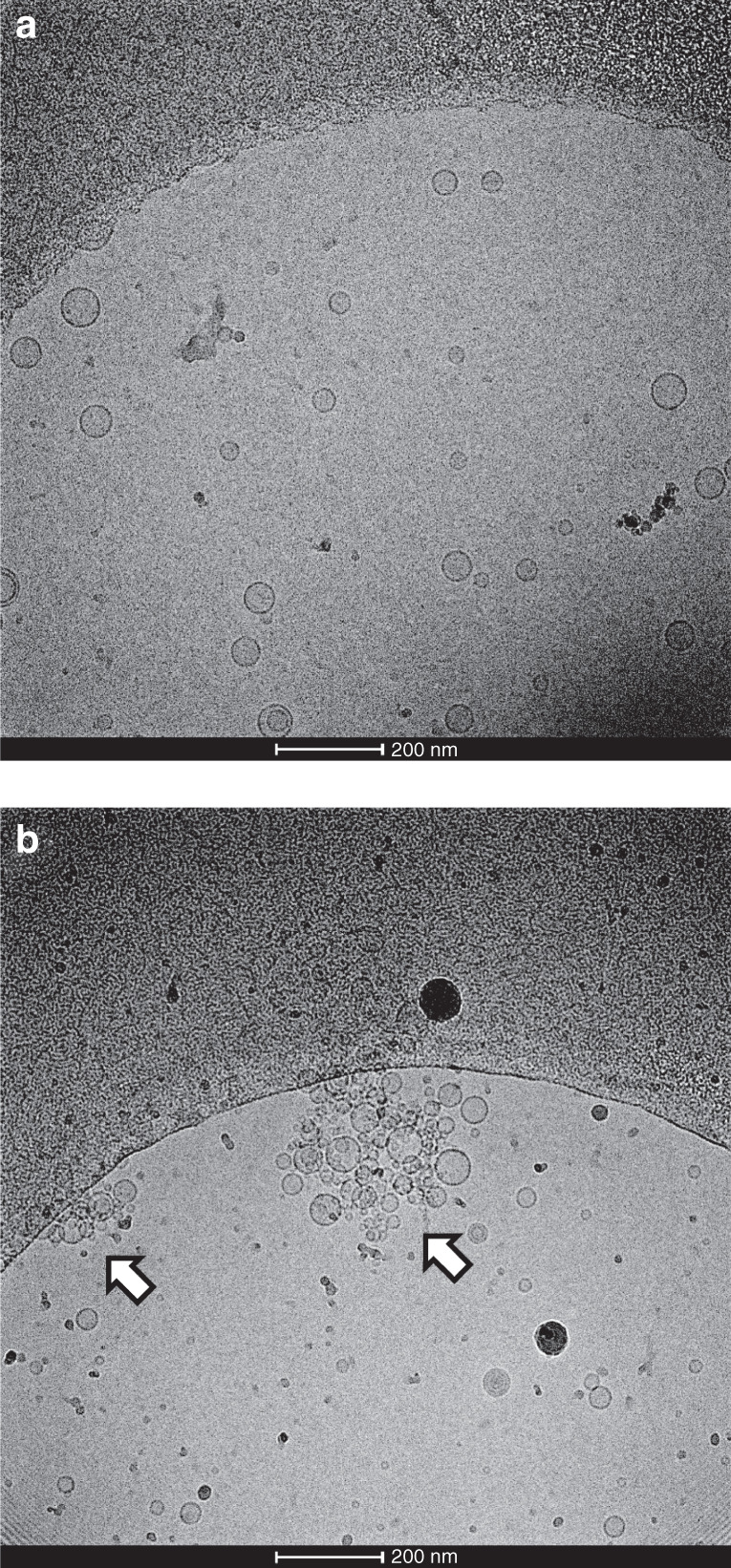
α-Syn induces liposome clustering. Representative TEM images of α-Syn with **a** DOPC vesicles and **b** 12% DOPS:DOPC (12%:88%) vesicles. The arrows indicate the vesicle clusters. The protein-to-lipid ratio was 1:500. The incubation time was 60 min

To illustrate how Ca^2+^ regulates vesicle clustering with α-Syn, we used transmission electron microscopy (TEM) to image lipid vesicles with α-Syn in the absence of Ca^2+^. Figure 2 shows representative TEM images of α-Syn with lipid vesicles composed of 1,2-dioleoyl-sn-glycero-3-phosphocholine (DOPC) only or DOPC plus 1,2-dioleoyl-sn-glycero-3-phospho-l-serine (DOPS). As shown in Fig. 2a, because α-Syn rarely binds to vesicles without anionic lipids, the 100% DOPC vesicles dispersed in the solution. By contrast, numerous DOPS:DOPC (12%:88%) vesicles formed clusters with α-Syn, which indicates that vesicle clustering requires α-Syn to bind to anionic lipid vesicles, as shown in Fig. 2b. However, TEM measurements cannot quantify the capability of vesicle clustering induced by α-Syn.

**Fig. 3:**
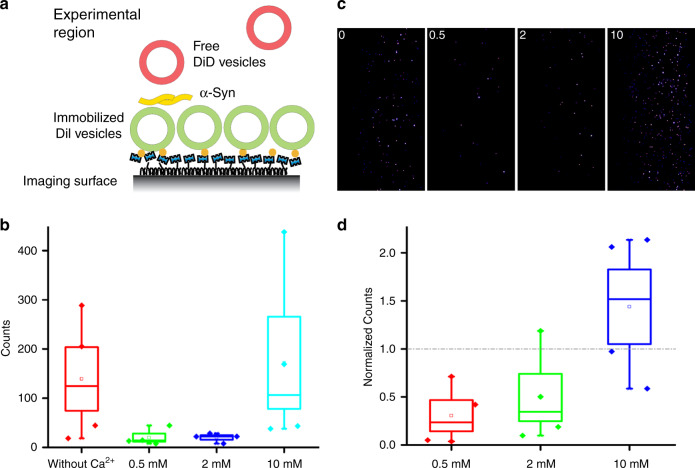
A single-vesicle clustering assay reveals the effect of various Ca^2+^ concentrations on the vesicle-clustering counts. **a** Illustration of the single-vesicle clustering assay. **b** The clustering counts. **c** The representative TIRF images of each channel, and **d** the normalized results of 12% PS vesicle clustering regulated by α-Syn. The case without Ca^2+^ was used as the control result

To quantify vesicle clustering induced by α-Syn, we performed a single-vesicle assay with two types of fluorescent dye to label different vesicles—free vesicles containing the fluorescent molecule DiI (DiIC18(3)) and immobilized vesicles containing the fluorescent molecule DiD (DiIC18(5)). The detection of vesicle clustering relied upon TIRFM (Fig. 3a), which can reveal individual vesicles clustered on the surface of immobilized vesicles. As shown in Fig. 3b, c, the concentrations of Ca^2+^ exerted a significant effect on vesicle clustering. At concentrations of 0.5 mM and 2 mM Ca^2+^, there were fewer vesicle clusters than in the control without Ca^2+^, which indicates that Ca^2+^ at concentrations of 0.5 mM and 2 mM inhibits the interaction of α-Syn with vesicles, as depicted in Fig. 3b, c. However, clusters at a concentration of 10 mM Ca^2+^ outnumbered those in the control, which suggests that higher concentrations of Ca^2+^ promote vesicle–vesicle interaction mediated by α-Syn. Due to surface variation, the baseline level of vesicle clustering differed significantly across different imaging surfaces; therefore, only experiments on the same imaging slide are directly comparable with absolute counts. To compare the results from different slides, we calculated the normalized value of the clustering counts (Fig. 3c) based on the original counts (Fig. 3b). A normalized value above or below 1 indicated the promotional or inhibitory effect of Ca^2+^, respectively. The normalized results shown in Fig. 3c reveal the concentration-dependent effect of Ca^2+^ on vesicle clustering with α-Syn, such that lower concentrations (i.e., 0.5 and 2 mM) of Ca^2+^ inhibited clustering, whereas a higher concentration (i.e., 10 mM) promoted it.

**Fig. 4:**
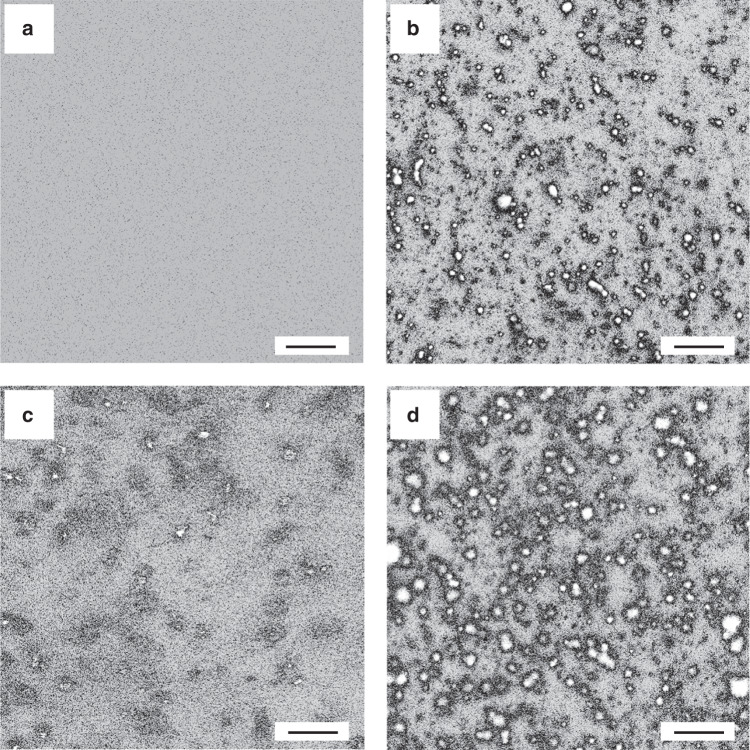
Vesicle-clustering images of 12% PS vesicles under varied conditions. **a** Vesicles only, **b** with α-Syn only, **c** with α-Syn and 0.5 mM Ca^2+^, and **d** with α-Syn and 10 mM Ca^2+^. Scale bars: 10 μm

To cross-check our results, we performed a bulk vesicle-clustering experiment in the presence of α-Syn and different concentrations of Ca^2+^. We incubated α-Syn with vesicles with 12% PS that were approximately 50 nm in diameter, and that had been labeled with the fluorescent molecule DiD. Although no significant aggregates appeared in the solution with vesicles only (Fig. 4a), vesicles in the α-Syn-containing solution formed some large aggregates, which confirmed that vesicle clustering is mediated by α-Syn, as shown in Fig. 4b. However, the level of aggregation was reduced when 0.5 mM Ca^2+^ was introduced (Fig. 4c), which demonstrates the inhibitory effect of 0.5 mM Ca^2+^ on vesicle clustering. After incubation of vesicles in 10 mM Ca^2+^ in the presence of α-Syn, an increasing number and size of aggregates formed, which shows that vesicle clustering is promoted by 10 mM Ca^2+^ (Fig. 4d). Such results confirmed the concentration-dependent effect of Ca^2+^ on vesicle clustering with α-Syn, such that lower concentrations (i.e., 0.5 and 2 mM) of Ca^2+^ inhibited clustering, whereas a higher concentration (i.e., 10 mM) promoted clustering.

### Ca^2+^ regulates vesicle clustering via its electrostatic interaction with lipid membranes

**Fig. 5:**
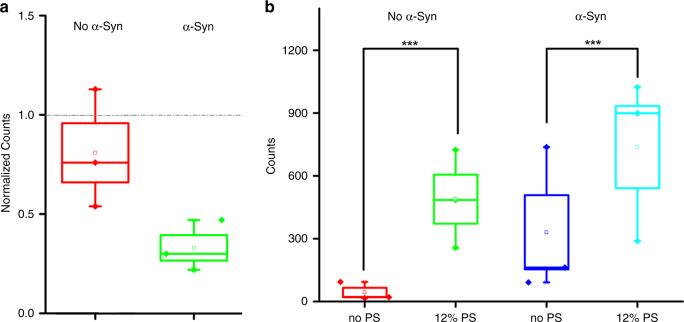
The effect of charged lipids (PS) on the clustering counts of lipid vesicles at **a** 0.5 mM and **b** 10 mM Ca^2+^. In **a**, the change is normalized to the number before adding 0.5 mM Ca^2+^

To gain insights into the mechanism by which Ca^2+^ regulates vesicle clustering, we performed vesicle-clustering experiments in the presence and absence of α-Syn with different concentrations of Ca^2+^. Moreover, to investigate whether electrically charged lipids affect the clustering interaction in the presence or absence of α-Syn, we prepared lipid vesicles with no electric charge (i.e., no PS) as a control group. Figure 5a shows the clustering counts of vesicles with no PS and with 12% PS in a low concentration of Ca^2+^ (i.e., 0.5 mM) with and without α-Syn. When α-Syn was absent, no significant difference in vesicle-clustering counts was observed between the vesicles without PS and those with 12% PS, which indicates that low concentrations of Ca^2+^ do not affect vesicle clustering in the absence of α-Syn. However, when α-Syn was present, clustering counts in vesicles with 12% PS were significantly less than those in vesicles without PS. Thus, the result shown in Fig. 5a suggests that vesicle clustering is mediated by α-Syn and inhibited by Ca^2+^ at a low concentration (i.e., 0.5 mM).

By contrast, at a high concentration of Ca^2+^ (i.e., 10 mM) without α-Syn, 12% PS increased the vesicle-clustering counts, regardless of whether α-Syn participated in the clustering process (Fig. 5b). The result suggests that Ca^2+^ can enhance vesicle clustering only at high concentrations with negatively charged lipids. The promotional role of Ca^2+^ at high concentrations (e.g., 10 mM) in vesicle clustering may occur via electrostatic interactions between Ca^2+^ and negatively charged lipids, as shown by earlier findings that multivalent ions may prompt ion bridges and tight coupling between lipid membranes^27^. Thus, whereas a high concentration of Ca^2+^ causes many Ca^2+^ to bind to lipids, and bound multivalent ions can bridge vesicles to form clusters, a low concentration of Ca^2+^ cannot supply sufficient Ca^2+^ to drive vesicles into close contact.

### Ca^2+^ inhibits the interaction between α-Syn and lipid membranes

**Fig. 6:**
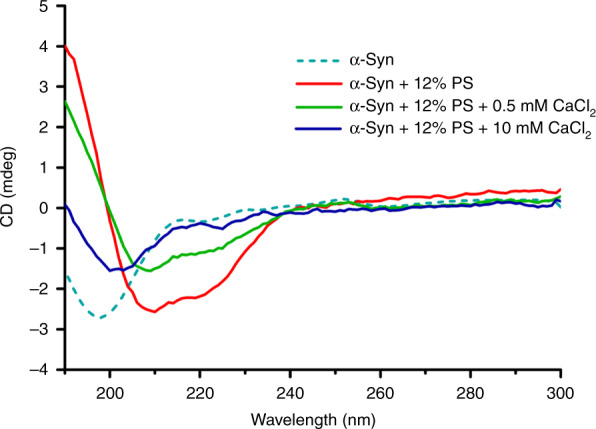
The CD spectra of α-Syn after mixing with 12% PS vesicles and varied Ca^2+^ concentrations. The group with only wild-type α-Syn was set as a control

Having observed the inhibitory effect of low concentrations of Ca^2+^ and the promotional effect of high concentrations, we recorded the circular dichroism (CD) spectra of α-Syn in CD buffer (20 mM of Na_2_HPO_4_/NaH_2_PO_4_ and 100 mM of NaF, pH 7.4), as shown in Fig. 6. The spectrum of the control group (i.e., free α-Syn only) had a minimum ellipticity of approximately 198 nm, which suggests that free α-Syn possesses a high percentage of random coil structures. After α-Syn was mixed with lipid vesicles containing 12% PS, two new characteristic minima appeared at 210–222 nm without any inverse peak in the vicinity of 198 nm, which demonstrates that highly unstructured α-Syn undergoes a conformational change into a typical secondary structure (i.e., α-helix) thought to be the stable conformation of lipid-bound α-Syn. Nevertheless, the intensity of the characteristic inverse peak at 210–222 nm decreased upon mixing with 0.5 mM Ca^2+^, which revealed that the content of the α-helix structure of lipid-bound α-Syn decreased. This result confirmed that the inhibitory effect of 0.5 mM Ca^2+^ on negatively charged vesicle clustering stemmed from the decreased amount of lipid-bound α-Syn. When 10 mM Ca^2+^ was applied to the lipid-bound α-Syn, the characteristic inverse peaks at 210–222 nm disappeared, and characteristic minima of random coil structures were observed at approximately 200 nm, which implies the nearly complete removal of lipid-bound α-Syn from the negatively charged lipid vesicles. The reason the restored peaks corresponding to a random coil shifted slightly compared with that of free α-Syn is probably that a large amount of Ca^2+^ bound to the removed α-Syn. Our CD spectrum experiments thus suggest that Ca^2+^, regardless of its concentration, inhibits the interaction between α-Syn and negatively charged lipid membranes.

**Fig. 7:**
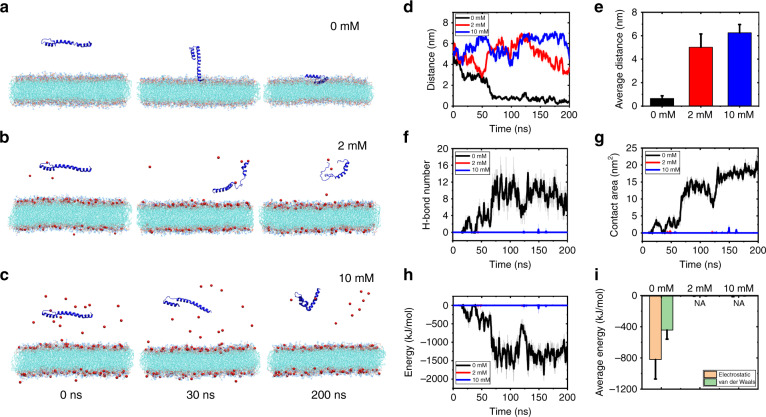
MD simulations of the N terminus of α-Syn binding with the lipid membrane with varied Ca^2+^ concentrations. **a**–**c** Snapshots of the N terminus of α-Syn binding with the lipid membrane with 0, 2, and 10 mM Ca^2+^, respectively. Ca^2+^ is represented by red dots, while Cl^−^ is omitted for clarity. **d** The distances versus simulation time, and **e** the average values between the N terminus of α-Syn and the surface of the lipid membrane. **f** The H-bond number and **g** the contact area between the N terminus of α-Syn and the lipid membrane. **h** The total interaction energy between the N terminus of α-Syn and the lipid membrane. **i** The average electrostatic and van der Waals interactions between the N terminus of α-Syn and the lipid membrane with varied Ca^2+^ concentrations. The average values in (**e**) and (**i**) were calculated by the simulation data in 100–200 ns

Speculating that the presence of Ca^2+^ affected α-Syn’s interaction with lipid membranes, and to confirm the interaction between α-Syn and lipid vesicles, we performed molecular dynamics (MD) simulations to study the interaction of α-Syn and lipid membranes mediated by Ca^2+^. The MD simulation method is a powerful approach to study MD and interactions. Previous studies have used MD simulations to study the conformations of α-Syn in membranes^28^, the dynamics of N-terminally acetylated α-Syn with lipids^29^, the influence of membrane curvature on α-Syn conformation^30^, and the effect of Ca^2+^ on different types of protein structures^31^. In particular, we tested the binding of α-Syn’s N-terminal domain (amino acids 1–60) to the anionic membrane in different concentrations of Ca^2+^. As shown in Fig. 7a, without Ca^2+^, the N terminus of α-Syn quickly bound to the membrane with negatively charged lipids, consistent with previous findings that α-Syn prefers to bind to anionic lipids via electrostatic interactions^32,33^. Snapshots of the details of α-Syn’s N-terminal binding to the membrane in the absence of Ca^2+^ can be found in Fig. S1. By contrast, when Ca^2+^ at concentrations of 2 mM and 10 mM was in the solution, the N terminus of α-Syn had no contact with the membrane, as shown in Fig. 7b–e, depicting the distance evolutions calculated and the average values of α-Syn’s N terminus to the lipid membrane, respectively, which confirmed the results suggesting that, without Ca^2+^, α-Syn’s N terminus is in close contact with the membrane. However, with Ca^2+^ at concentrations of 2 and 10 mM, α-Syn’s N terminus clearly distances itself from the membrane’s surface. Figure 7f, g shows that H bonds and contacts formed between α-Syn’s N terminus and lipid membranes in the absence of Ca^2+^, whereas none of them formed in the presence of Ca^2+^. Figure 7h, i illustrates the interaction energies between α-Syn’s N terminus and lipid membranes, confirming that electrostatic interaction is the primary driving force of their binding, and that Ca^2+^ that competitively bound to negatively charged lipids inhibits the interaction between α-Syn and the membranes. Consistent with the CD experiments (Fig. 6) and previous findings^16,17^, the MD results showed that Ca^2+^ binds to membranes with negatively charged lipids that block α-Syn’s binding to the membranes.

## Discussion

α-Syn, an intrinsically disordered protein in aqueous solution, is abundantly expressed throughout the central nervous system. Recent studies have suggested that α-Syn binds to small synaptic vesicles with highly curved membranes via a conserved lipid-binding domain, senses and regulates the curvature of the membranes, and may be involved in both synaptic vesicle trafficking and the exo- and endocytosis of synaptic vesicles^9–11^. Furthermore, other research has proposed that α-Syn can act as a bridge between membranes in which two helices bind to two synaptic vesicles and, in turn, cause vesicles to cluster^15^. Similarly, another recent study showed that α-Syn amyloid oligomers, acting as multivalent nanoparticles, can cause close contact and even hemifusion in negatively charged vesicles^19^. Because researchers have also proposed that Ca^2+^ can regulate the interaction between α-Syn and lipid membranes^16–18^, we performed single-vesicle imaging to study vesicle clustering mediated by α-Syn and the regulation of Ca^2+^.

Our results clearly indicate that α-Syn can act as an intermediate in vesicle clustering by binding to negatively charged lipid vesicles, as shown in Fig. 2. A single-vesicle clustering assay revealed that the effect of Ca^2+^ in vesicle clustering with α-Syn varies by concentration; at low concentrations, Ca^2+^ inhibited vesicle clustering with α-Syn (Fig. 3), which can be attributed to the mechanism by which the binding of Ca^2+^ to lipids blocks the interaction of α-Syn with vesicles, such that α-Syn cannot mediate vesicle clustering. This result is consistent with Zhang et al.’s^16,17^ finding that Ca^2+^ competitively binds to anionic lipids and thereby induces the dissociation of α-Syn from membranes. At the same time, with more Ca^2+^ in the solution—that is, at high concentrations of Ca^2+^—Ca^2+^ promotes vesicle clustering. Ca^2+^ without α-Syn also enhanced vesicle clustering at high concentrations of Ca^2+^ (Fig. 5). However, CD spectroscopy experiments and MD simulations indicated that Ca^2+^ inhibited the interaction of α-Syn’s N terminus with vesicles regardless of the Ca^2+^ concentration (Figs. 6 and 7). The promotional role of high concentrations of Ca^2+^ in vesicle clustering may occur via the electrostatic interaction of Ca^2+^ and negatively charged lipids, which would corroborate previous findings that multivalent ions can cause ion bridges and tight coupling between lipid membranes ^27^.

Our results also suggest that the mechanism of Ca^2+^ in regulating vesicle clustering varies with the Ca^2+^ concentration. At low concentrations, Ca^2+^ competes with α-Syn to bind to anionic lipids, which causes α-Syn to dissociate from membranes such that vesicle clustering mediated by α-Syn does not occur. By contrast, although Ca^2+^ may inhibit α-Syn’s interaction with membranes, a high concentration of Ca^2+^ can supply enough multivalent ions to drive vesicles into close contact and, in turn, form clusters.

## Conclusion

α-Syn has been shown to interact with isolated synaptic vesicles via its vesicle-binding domain, which results in the clustering of synaptic vesicles, and Ca^2+^ can regulate vesicle clustering mediated by α-Syn. Using single-vesicle imaging, we studied how Ca^2+^ regulates vesicle clustering mediated by α-Syn and the possible underlying mechanism. We showed not only that Ca^2+^ plays a critical role in vesicle clustering induced by α-Syn, but also that different concentrations of Ca^2+^ exerted different effects on vesicle clustering mediated by α-Syn via different mechanisms. Whereas a high concentration of Ca^2+^ promoted vesicle clustering in an α-Syn-independent manner via the electrostatic interaction of Ca^2+^ and negatively charged lipids, low concentrations of Ca^2+^ inhibited vesicle clustering in an α-Syn-dependent manner, in which Ca^2+^ competitively bound to anionic lipids, blocking α-Syn’s interaction with lipid membranes. Our bulk clustering experiments also confirmed the different effects of low and high concentrations of Ca^2+^ on vesicle clustering mediated by α-Syn, while our CD experiments and MD simulations confirmed the underlying mechanisms, namely, that Ca^2+^ removes the N terminus of α-Syn from the anionic membrane and inhibits vesicle clustering mediated by α-Syn. Our work suggests that Ca^2+^ and negatively charged lipids play critical roles in vesicle clustering, and provides new insights into how α-Syn regulates synaptic vesicle trafficking and synaptic transmission.

## Methods

### Preparation of PEGylated slides for single-vesicle fluorescence imaging

To study vesicle clustering, samples were loaded into a chamber assembled from quartz slides and glass coverslips. It is essential to have clean surfaces for single-vesicle fluorescence imaging. First, the slides and coverslips were rinsed and sonicated using Milli-Q H_2_O, acetone, 1 M KOH solution, and methanol, sequentially. After that, the slide and coverslip surfaces were burned for more than 1 min and 2 s, respectively, to remove potential residues. To prevent nonspecific adsorption, the surfaces were PEGylated to achieve passivation, which included three steps, i.e., silanization, biotinylation, and washing. In brief, the slides and coverslips with clean surfaces were first incubated in amino silane ((3-(2-aminoethylamino) propyl) trimethoxysilane) solution (100 ml of methanol, 5 ml of acetic acid, and 1 ml of amino silane) for 10 min, sonicated for 1 min, and incubated for 10 min again. Each slide or coverslip was then blown dry from the edge using nitrogen gas after washing with Milli-Q H_2_O followed by methanol. Finally, 100 μl of reaction solution (120 mg of mPEG, ~4 mg of biotin–PEG, and 700 μl of 0.1 M sodium bicarbonate solution) was placed onto the imaging surface of the slide. Each slide was covered by one clean coverslip to be a set, which was incubated overnight and then rinsed with Milli-Q H_2_O, dried, and stored at −20 °C for further use.

### Preparation of lipid vesicles

We used two types of lipid vesicles with fluorescent dyes, i.e., DiI (DiIC18(3)) and DiD (DiIC18(5)), for single-vesicle clustering experiments. Before mixing the lipid reagents, the glass syringe was washed three times with chloroform. The lipid components were added into the glass tube wrapped with a piece of foil with a small hole at the top. For preparation of 12% PS vesicles, 103.8 μl of DOPC (1,2-dioleoyl-sn-glycero-3-phosphocholine), 29.8 μl of DOPE (1,2-dioleoyl-sn-glycero-3-phosphoethanolamine), 48.6 μl of DOPS (1,2-dioleoyl-sn-glycero-3-(phospho-l-serine)), 6.4 μl of Bio-PE (1,2-dioleoyl-sn-glycero-3-phosphoethanolamine-N-(biotinyl)) (only for DiI vesicles) and fluorescent dye (100 μl of DiI or DiD) were included in the composition required. The lipid composition of the no-PS vesicles was similar to that of 12% PS vesicles, except for the absence of DOPS. To form a thin lipid film, the mixture in the tube was subjected to vacuum in a ball container and rotated every 30 min until the organic solvent was completely evaporated. Subsequently, 500 μl of HEPES buffer (25 mM HEPES and 100 mM NaCl, pH 7.4) was added to hydrate the lipid film. Finally, the solution was frozen and thawed at least 5–6 times to obtain vesicles that were filtered through a membrane with 50-nm pores. The diameter distribution of 40–50-nm liposomes was previously measured by TEM^34^. The dispersed vesicles were stored at −80 °C for further experiments.

### Single-vesicle clustering experiments

The no-PS or 12% PS vesicles with DiI were first immobilized on the imaging surface of PEGylated quartz slides via interaction between biotin and NeutrAvidin. The imaging area should be fully covered by DiI vesicles and produced a homogeneous distribution. After incubation for 30 min and buffer change, wild-type α-Syn was injected into the sample chamber and incubated for 15 min. The channel without supply of α-Syn was set as a control. The DiD vesicle solution corresponding to the immobilized DiI vesicles at the required protein–lipid ratio was injected into each channel after buffer exchange to remove unbound α-Syn. To investigate the effects of Ca^2+^ or other divalent ions on vesicle clustering, Ca^2+^ or other divalent ions were mixed with DiD vesicles at different concentrations and injected into the sample chambers immediately. Before imaging under wide-field TIRFM, samples in the channels were incubated for 20 min and washed with HEPES buffer three times to remove uncombined vesicles.

DiI and DiD vesicles were detected and imaged after the dyes were excited by a green laser (532 nm) and a red laser (633 nm), respectively. The CCD camera and smCamera program were used to acquire and analyze the images, respectively. The number of vesicles clustered was determined by counting the DiD vesicles whose fluorescent spots were shown in the acceptor channel on each image (45 × 90 μm). Approximately, 15 random locations were imaged and analyzed for each sample channel. The compared results are expressed as the mean ± standard deviation. One-way analysis of variance (ANOVA) with Tukey’s test was used to determine the statistical significance among different groups. When *P* < 0.001, the difference was considered extremely significant (***).

### Bulk clustering

To investigate bulk vesicle clustering, the DiD vesicles were dispersed and diluted in 3 wt% mPEG (Mw: 5000) solution to 2 μM. The 1:500 protein–lipid ratio was used to study the bulk clustering in the presence of 4 nM α-Syn. A 10 mM or 0.5 mM Ca^2+^ solution was added to the mixture of α-Syn and lipid vesicles to observe the effect of Ca^2+^. The mixture was incubated for more than 20 min before imaging the samples excited by red laser light with confocal microscopy. Imaging was performed using 35-mm glass-bottom dishes (MatTek).

### CD spectroscopy

The lipid composition of the 12% PS vesicles used for the CD experiments includes 43 mol% DOPC, 45 mol% DOPE, and 12 mol% DOPS. Dried lipid films were rehydrated in deionized water to a concentration of 5 mM. Vesicles were formed after at least six freeze (liquid N_2_)–thaw (37 °C water bath) cycles. Before use, the vesicles were sonicated three times in low-power mode. Each sonication was performed for 5 min. α-Syn was dissolved in Tris-HCl buffer (25 mM Tris-HCl and 10 mM NaCl, pH 7.4) to form a protein solution of 2.5 mg/ml, which was dialyzed in 300 ml of CD buffer overnight before use. The final protein solution for detection was 10 μM. The protein-to-lipid ratio in the mixture of α-Syn and lipids used for each test was 1:100. CaCl_2_ (0.5 mM or 10 mM) dissolved in Tris-HCl buffer was mixed with the corresponding lipid-bound protein solution. The secondary structure of lipid-free or lipid-bound α-Syn was determined by CD spectroscopy (Aviv 215 CD spectrophotometer). Wavelength scans were taken in 0.5-nm steps with a 3-s averaging time and 0.333-s settling time. Spectra were collected from 300 nm to 190 nm at 25 °C in a 0.1-mm path-length quartz cuvette containing samples. The baseline was corrected by the blank (buffer only). Each spectrum was averaged from three repeats. The corrected signal data of each CD spectrum were smoothed with the adjacent-averaging method and then plotted using Origin8.5.

### Preparation of MD simulation models

The lipid bilayers were generated by the Membrane Builder online service CHARMM-GUI^35,36^. Here, the lipid composition was adopted as DOPC:DOPE:DOPS with a ratio of 45%:43%:12%. DOPC, DOPE, and DOPS are abbreviations for 1,2-dioleoyl-sn-glycero-3-phosphocholine, 1,2-dioleoyl-sn-glycero-3-phosphoethanolamine, and 1,2-dioleoyl-sn-glycero-3-phospho-L-serine, respectively. To reduce the computational demand, the membrane was built as a flat membrane. To mimic the packing density of lipids in a curved membrane, we applied tension to the flat membrane by $\sigma =\langle {L_{z}/2[ {p_{zz} - 1/2( {p_{xx} + p_{yy}} )}]} \rangle$, where *σ* is the membrane tension, *p*_*ij*_ is the *ij* component of the pressure, *L*_*z*_ is the box length along the *z* direction, and <·> represents the time average^37–39^. According to the packing density of lipids in vesicles (e.g., ~1.19–1.35 lipids per square nanometer for a vesicle with an ~20-nm diameter^40,41^), we applied *p*_*zz*_ = 1 bar, *p*_*xx*_ = *p*_*yy*_ = −28 bar for 15 ns to achieve a packing density of ~1.28 lipids per square nanometer of the membrane, as shown in Fig. S2. The system was solvated in an ~15 × 15 × 12 nm^3^ TIP3P^42^ water box, with ~65,000 water molecules and an appropriate number of sodium ions added to neutralize the system.

According to a previous study, Ca^2+^ in solution will significantly bind to the membrane with negatively charged lipids^27,43^. To prepare the membrane model with 2 mM and 10 mM Ca^2+^, we gradually added CaCl_2_ molecules to the solution, and Ca^2+^ bound to the membrane, as shown in Fig. S3. Until the concentration of Ca^2+^ in solution was static at 2 mM and 10 mM for 20 ns, the structures were extracted for simulations of α-Syn binding, as shown in Fig. S3. The structure of α-Syn was adopted from PDB: 1XQ8^13^. To reduce the computational demand, only the N terminus of α-Syn (i.e., residues 1–60) was used in the simulations.

### MD simulation method

All of the simulations were performed using the GROMACS package^44^ with the CHARMM36 force field^45^. Periodic boundary conditions were applied, and the temperature was coupled to 310.25 K with the V-rescale algorithm^46^. To maintain the packing density of lipids, the pressure was coupled by the Parrinello–Rahman method as *p*_*zz*_ = 1 bar, *p*_*xx*_ = *p*_*yy*_ = −28 to apply membrane tension^47^. The LINCS algorithm was applied to constrain the covalent bonds with H atoms^48^. The time step of the simulations was 2.0 fs. The particle mesh Ewald method was used to calculate the long-range electrostatic interactions^49^. The cutoff of the nonbonded interactions was set to 12 Å. All graphics and visualization analyses were processed using the VMD package ^50^.

## Supplementary information

Supporting Materials
