# Supplementary material for: Single-vesicle imaging quantifies calcium’s regulation of nanoscale vesicle clustering mediated by α-synuclein
Source: Microsyst Nanoeng. 2020 Jun 29;6:38. doi: 10.1038/s41378-020-0147-1 (PMC8433175; doi:10.1038/s41378-020-0147-1)
Supplement: Supplementary file 1 — Supporting Materials [file 41378_2020_147_MOESM1_ESM.docx]

**Supplementary materials**

**Single-vesicle imaging quantifies calcium’s regulation of nanoscale vesicle clustering mediated by α-synuclein**

Bin Cai^1*^, Jie Liu^2*^, Yunfei Zhao^3*^, Xiangyu Xu^2^, Bing Bu^4^, Dechang Li^5^†, Lei Zhang^6^†, Wei Dong^3^†, Baohua Ji^5, 7^, Jiajie Diao^1^†

1. Department of Cancer Biology, University of Cincinnati College of Medicine, Cincinnati, OH 45267, USA.
2. Biomechanics and Biomaterials Laboratory, Department of Applied Mechanics, Beijing Institute of Technology, Beijing 100081, China.
3. Key Laboratory of Medical Electrophysiology of Ministry of Education and Medical Electrophysiological Key Laboratory of Sichuan Province, Institute of Cardiovascular Research, Southwest Medical University, Luzhou, Sichuan 646000, China.
4. Institute of Biomedical Engineering and Health Sciences, Changzhou University, Changzhou, Jiangsu 213164, China.
5. Institute of Applied Mechanics, Department of Engineering Mechanics, Zhejiang University, Hangzhou 310027, China.
6. Key Laboratory for Nonequilibrium Synthesis and Modulation of Condensed Matter (Ministry of Education), School of Science, Xi’an Jiaotong University, Xi’an 710049, China.
7. Beijing Advanced Innovation Center for Biomedical Engineering, Beijing 100191, China


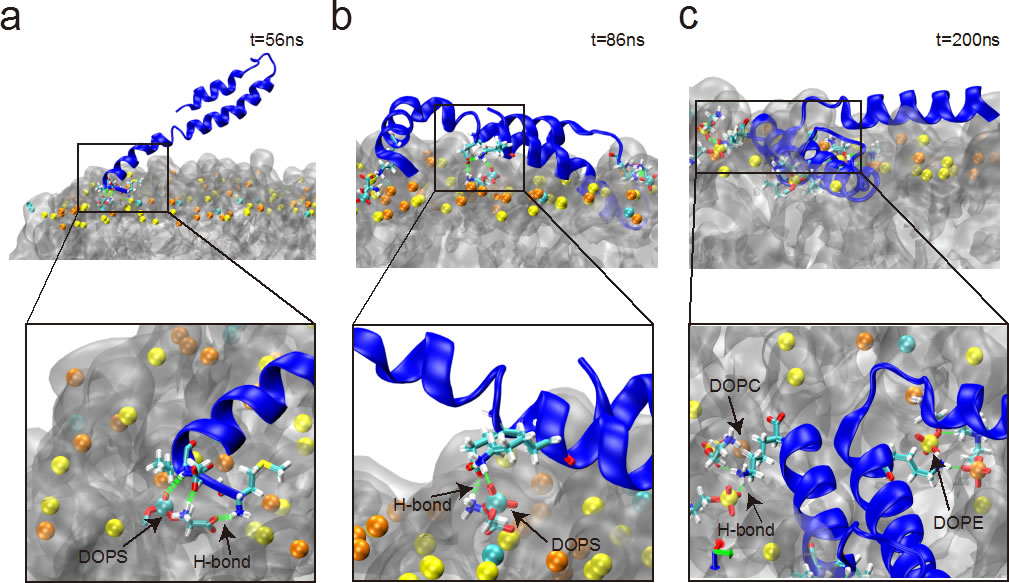


**Fig. S1.** Detailed snapshots of α-Syn’s N-terminal binding to membrane in the absence of Ca^2+^.

**Fig. S2.** The packing density of lipids with membrane tension.


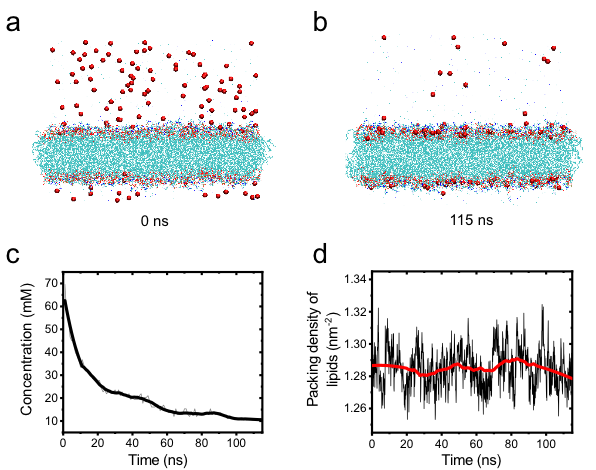


**Fig. S3.** Preparation for the membrane model with 10 mM Ca^2+^ ions. (**a**) The initial structure of the system. The red dots represented the Ca^2+^ ions. The Cl- ions were not shown for clarify. (**b**) After 115 ns simulation, most of the Ca^2+^ ions bound to the membrane. (**c**) The concentration of Ca^2+^ ions in solution and (**d**) the packing density of lipids when Ca^2+^ ions bind to the membrane.
